# Artificial Neural Network for Atrial Fibrillation Identification in Portable Devices

**DOI:** 10.3390/s20123570

**Published:** 2020-06-24

**Authors:** Daniele Marinucci, Agnese Sbrollini, Ilaria Marcantoni, Micaela Morettini, Cees A. Swenne, Laura Burattini

**Affiliations:** 1 Department of Information Engineering, Università Politecnica delle Marche, via Brecce Bianche 12, 60131 Ancona, Italy; daniele.marinucci90@gmail.com (D.M.); a.sbrollini@pm.univpm.it (A.S.); i.marcantoni@pm.univpm.it (I.M.); m.morettini@univpm.it (M.M.); 2 Cardiology Department, Leiden University Medical Center, P.O. Box 9600, 2300 RC Leiden, The Netherlands; c.a.swenne@lumc.nl

**Keywords:** atrial fibrillation, machine learning algorithms, artificial neural networks, portable devices

## Abstract

Atrial fibrillation (AF) is a common cardiac disorder that can cause severe complications. AF diagnosis is typically based on the electrocardiogram (ECG) evaluation in hospitals or in clinical facilities. The aim of the present work is to propose a new artificial neural network for reliable AF identification in ECGs acquired through portable devices. A supervised fully connected artificial neural network (RSL_ANN), receiving 19 ECG features (11 morphological, 4 on F waves and 4 on heart-rate variability (HRV)) in input and discriminating between AF and non-AF classes in output, was created using the repeated structuring and learning (RSL) procedure. RSL_ANN was created and tested on 8028 (training: 4493; validation: 1125; testing: 2410) annotated ECGs belonging to the “AF Classification from a Short Single Lead ECG Recording” database and acquired with the portable KARDIA device by AliveCor. RSL_ANN performance was evaluated in terms of area under the curve (AUC) and confidence intervals (CIs) of the received operating characteristic. RSL_ANN performance was very good and very similar in training, validation and testing datasets. AUC was 91.1% (CI: 89.1–93.0%), 90.2% (CI: 86.2–94.3%) and 90.8% (CI: 88.1–93.5%) for the training, validation and testing datasets, respectively. Thus, RSL_ANN is a promising tool for reliable identification of AF in ECGs acquired by portable devices.

## 1. Introduction

Arrhythmias are among the most common cardiac disorders that can cause severe and sometimes fatal complications, even when asymptomatic [1,2]. Among the different kinds of serious cardiac arrhythmias, atrial fibrillation (AF) is the most common, affecting 1–2% of the worldwide population [3]. AF is associated with a high morbidity (especially stroke and heart failure) and mortality. Mortality (per 100,000 population), in particular, has shown an increasing trend with time; from 1990 to 2010 it increased from 0.8 to 1.6 in men, and from 0.9 to 1.7 in women, with peaks in developed countries reaching 2.7 and 2.4, respectively [3]. Thus, AF represents, worldwide, a significant public health problem with huge socio-economic repercussions.

**Figure 1:**
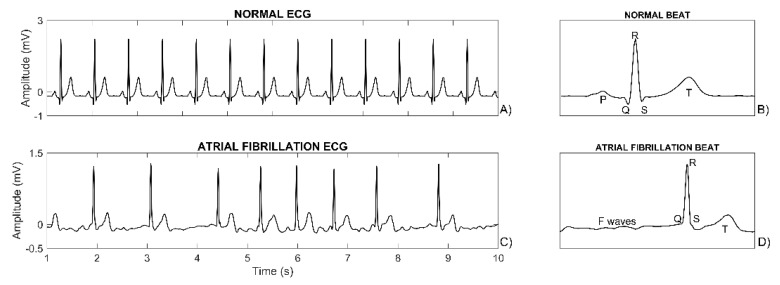
Panel (**A**) shows a normal pseudo-periodic electrocardiogram (ECG) tracing. Panel (**B**) shows a normal beat, constituted by a P wave (the smallest wave), a QRS complex (with R being the highest wave) and a T wave. Panel (**C**) shows an ECG tracing with atrial fibrillation (AF) and thus increased heart rate variability (HRV). Panel (**D**) shows a beat during AF with F waves but no P wave.

AF is a supraventricular arrhythmia characterized by uncoordinated continuous atrial electrical activation, causing the deterioration of atrial functionality. In normal conditions, the contraction of the heart is initiated by an electrical impulse that, after having been generated by the sino-atrial node, propagates through all atrial myocardial cells, causing their electrical depolarization and mechanical contraction followed by the electrical repolarization and mechanical relaxation. Successively, the electrical impulse reaches the atrioventricular node, in which it is slowly conducted before propagating through all ventricular myocytes, causing their ventricular depolarization and contraction and subsequent repolarization and relaxation. The electrical phenomena associated with the propagation of this impulse through the heart result in typical waves of the electrocardiogram (ECG) measured at the body surface. Normally, the ECG is a pseudo-periodic signal (Figure 1A) constituted by the repetition of a pattern showing a sequence of typical waves (Figure 1B): the P wave, which reflects the atrial depolarization; the QRS complex, which reflects the ventricular depolarization and hides the atrial repolarization; and the T wave, which reflects the ventricular repolarization. In AF, the sino-atrial node is overruled by the continuous fibrillatory activity and is no longer able to provide its pseudo-periodic impulse, so the heart rhythm becomes irregular (Figure 1C) and the impulse propagates though the atria following chaotic pathways [4]. However, once the impulse reaches the atrioventricular node and finds it not refractory, the impulse normally propagates through the ventricles. Combination of the AF random nature and the complex conduction/blocking properties of the atrioventricular node generates an irregular heart rate. These abnormalities of the electrical activity of the heart are reflected in the ECG that is no longer a pseudo-periodic signal but, rather, shows a high level of heart-rate variability (HRV) (Figure 1C). The P wave is no longer present; instead, continuous fibrillatory waves, also called F waves, are seen as rapid low amplitude oscillations that reflect the continuous uncoordinated atrial depolarization (Figure 1D).

AF diagnosis is typically ECG-based and is usually made by a cardiologist, possibly supported by computerized applications [5,6,7,8,9,10,11,12,13,14,15,16,17,18], in hospitals or in clinical facilities. However, traditional medical ECG devices, even when used out-of-the-hospital (such as the Holter ECG recorders), are coupled to a limited amount of people, who are symptomatic or have cryptogenic stroke and, hence, for whom there is an indication for long-term monitoring. But, due to the sneaky and oftentimes asymptomatic way AF develops, a large-scale monitoring would be preferable, especially in the population above a certain age. The use of wearable devices (such as watches, patches and bands) and portable devices (such as smartphone and tablets) is becoming more and more common among the entire population worldwide. The modern devices are able to record the ECG and thus have opened the possibility to remotely monitor AF on a plethora of individuals. However, in order to be useful in the preventive diagnosis of AF, they have to be associated with a reliable diagnostic software. As a result, several algorithms for automatic detection of AF have been proposed in the literature [5,6,7,8,9], several of which are based on machine and deep learning approaches [10,11,12,13,14,15,16,17,18]. Most of them claim very high performances but, when critically analyzed, show some common limitations. Firstly, performances of some algorithms for AF identification have been tested only against sinus rhythm [5,6,7,8,9,10,11,13,14,15], without considering the main confounders that are the level of noise affecting ECGs and the presence of other kinds of arrhythmias [12,16,17,18]. Secondly, most algorithms only rely on HRV to identify AF [5,6,7,8,9,10,13,14], despite high HRV being also associated with many other arrhythmias (not AF-specific) [19,20] and AF being also associated with absence of electrocardiographic P wave and presence of electrocardiographic F waves. Finally, some algorithms have been tested only on ECGs recorded by traditional medical devices [11,12,13,14,15,16,18] and not by modern wearable or portable devices; thus, their applicability to the latter remains to be demonstrated.

The aim of the present work is to propose a new artificial neural network (ANN) for a reliable identification of AF based on several input ECG features and to test it on ECG recordings acquired through portable devices, and thus typically affected by noise, made in healthy subjects and in cardiac patients exhibiting various types of abnormal cardiac rhythms. To this aim, a supervised fully connected artificial neural network was created using the repeated structuring and learning procedure [21] and tested on the “AF Classification from a Short Single Lead ECG Recording” database [19] by Physionet [22], consisting of thousands of short single-lead ECG recordings acquired with the portable KARDIA device by AliveCor [19].

## 2. Materials and Methods

### 2.1. Study Datasets

Data belong to the “AF Classification from a Short Single Lead ECG Recording” database by Physionet [19,22] (https://physionet.org). They include 8244 single lead ECGs (typically Einthoven lead I), collected with the portable KARDIA device by AliveCor (https://www.alivecor.com). ECG duration ranges from 9 s to 61 s (average: 33 s) and the sampling rate is 300 Hz. All ECG recordings were manually annotated by an expert as showing AF rhythms (738 recordings), normal rhythms (5050 recordings) or other rhythms (different from AF and normal rhythms, such as premature ventricular contraction; 2456 recordings) [19,22]. For the scope of this paper, these ECG recordings were classified into two reference classes, the AF class (738 recordings) and the non-AF class (7506 recordings).

All ECGs were characterized in terms of signal-to-noise ratio (SNR) (in dB, where the signal and noise amplitudes were defined as maximum signal amplitude and 4 times signal standard deviation, respectively) and submitted to an automatic algorithm for R-peak detection [23]. Only ECGs for which at least three consecutive R peaks could be identified were accepted for feature extraction and AF identification. Specifically, only the accepted ECGs were considered and grouped into three datasets, the training dataset, the validation dataset and the testing dataset. The training dataset and the validation dataset, including 55% and 15% of accepted ECGs, respectively, were used to create the ANN for AF identification, while the testing dataset, including the remaining 30% of accepted ECGs, was used to evaluate the created ANN performance. In all datasets, the prevalence of subjects in AF and non-AF classes was maintained unaltered.

### 2.2. ECG Processing and Feature Extraction

Initially, each ECG was prefiltered with a 6th order bidirectional Butterworth bandpass filter (cutoff frequencies of 0.5 Hz and 45 Hz) and R-peak positions were identified [23]. Then, several different signal processing steps were applied to obtain a set of 19 features from each ECG, 11 morphological features, 4 F-waves features and 4 HRV features. For an interpretive approach, the features were selected to include all those on which the criteria for AF diagnosis rely, namely P-wave disappearance, F-waves appearance and HRV increment, possibly quantified with different methods.

The 11 morphological features were extracted from the median ECG beat (MECGB), obtained as the median of the *n* (with *n* being the number of beats in the recordings) ECG segments included between 250 ms and 450 ms before and after each R peak, respectively. Specifically, the following 6 standard landmarks [24] were identified: P_p_ (position of the absolute maximum of │MECGB│ to the left of the R wave; it corresponds to the P-peak position in the presence of the P wave or to the highest oscillation position in the presence of F waves); R_p_ (position of the absolute maximum of │MECGB│; it corresponds to the R-peak position); T_p_ (position of the absolute maximum of │MECGB│ to the right of the R wave; it corresponds to the T-peak position); QRS_on_ (position of the point where the MECGB derivative changes its sign for the second-to-last time before R_p_; it corresponds to the QRS-onset position); QRS_off_ (position of the point where the MECGB derivative changes its sign for the second time after R_p_; it corresponds to the QRS-offset position or J point); and T_off_ (position of the point where the MECGB derivative changes its sign for the first time after the T_p_; it corresponds to the T-offset position). Using these 6 landmarks, 11 morphological features, 5 time intervals (namely P_p_R_p_, P_p_QRS_off_, QRS_on_QRS_off_, QRS_on_T_off_ and QRS_off_T_off_) and 6 amplitudes (namely AP, AQRS_on_, AQRS, AQRS_off_, AT and AQRS/AP), are computed as described in Table 1. All amplitude features are computed with respect to baseline level identified 80 ms before R_p_ [25].

The 4 F-waves features are based on the power spectral density estimation of the residual ECG obtained by subtracting the dominant ECG waveform obtained using the segmented beat modulation method [26,27], from the original ECG. Specifically, the F-waves frequency ratio (FWFR) (dimensionless); was computed as the ratio between the spectral area in the F-waves frequency band (4–10 Hz) and the total spectral area [27]. Since four different methods were used to estimate the power spectral density, 4 FWFR values (namely FWFR_FFT_, FWFR_WLC_, FWFR_YWK_, and FWFR_THM_,) were obtained as described in Table 1.

Finally, 4 HRV features (namely MRR, SDRR, RMSRR, and PRR50) were obtained from the RR interval series [28] as described in Table 1.

### 2.3. Artificial Neural Network Construction

The iterative repeated structuring and learning (RSL) procedure [21] was used to create a supervised fully connected artificial neural network (RSL_ANN). Details about the RSL procedure can be found in [21]. In the present study, RSL_ANN was designed according to the following specifications: (a) the input layer consists of 19 neurons (one for each extracted feature), the output layer consists of one neuron that provides a value between 0 and 1, with 0 representing the non-AF class and 1 representing the AF class, and all other neurons had weights and biases between −1 and +1 and a sigmoid activation function; (b) optimization was done with the scaled-conjugate-gradient algorithm [29]; (c) to avoid overfitting, the validation-based early stopping criterion was used [30]; and (d) the AF and non-AF classes were weighted according to the inverse of their prevalence in order to compensate their disproportionality [31]. The procedure dynamically alternated structuring and learning phases. The primitive RSL_ANN (initially composed of a neuron in a hidden layer) was upgraded in different alternatives according to the following rules: each alternative presented only an additional neuron in an existing layer or in a new layer; the number of neurons in a layer could not be larger than the number of neurons in the previous layer; the maximal number of layers was three; and initialized weights and bias of the additional neuron had to improve RSL_ANN performance after only one epoch. If one rule was not fulfilled, the alternative was not acceptable. Then, all alternatives were learnt, and their validation errors were compared with the validation error of the primitive RSL_ANN. The RSL_ANN with the smallest validation error was considered as the new primitive RSL_ANN, and the procedure started anew. The stopping criteria were the following: there were no acceptable alternatives; the same alternative was confirmed as primitive for 10 consecutive times; or there were no misclassifications in both training and validation datasets. When one of the stopping criteria occurred, the primitive RSL_ANN was also the final RSL_ANN. In order to avoid dependency from initialization, 100 different RSL_ANNs were created by considering 100 different initializations. The optimal RSL_ANN was selected as the one showing the smallest validation error.

### 2.4. Statistics

Feature distributions over classes were described in terms of 50th [25th;75th] percentiles in all datasets and compared using the Wilcoxon ranksum test for equal medians. Statistical significance (*p*-value) was set at 0.05. RSL_ANN performance was evaluated by computing the receiver operating characteristic (ROC) curve from which area under the curve (AUC) and associated 95% confidence intervals (CIs) were computed. Sensitivity (Se) and specificity (Sp) were eventually determined for two specific operating points on the ROC curve of the testing dataset. The first operating point (Case 1) was that for which Se equals Sp; the second operating point (Case 2) was that for which Sp is set at 75% and Se is computed accordingly.

## 3. Results

Out of 8244 ECGs available in the Physionet “AF Classification from a Short Single Lead ECG Recording” database, 8028 (97.4%) were accepted for the study while the remaining 216 (2.7%) were rejected. Accepted ECGs were characterized by a SNR significantly higher than rejected ones (3.7[1.2;4.3] dB vs. 0.1[−2.5;2.5] dB, respectively; *p*-value < 0.05). Table 2 shows accepted ECGs grouped into training, validation and testing datasets.

Feature distributions over datasets are reported in Table 3. Most features (15 out of 19) were found to be significantly different when statistically comparing the subjects in the AF and non-AF classes in all datasets.

**Figure 2:**
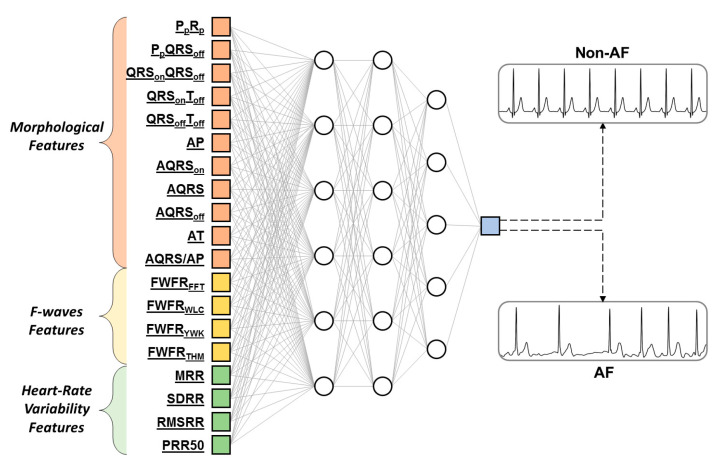
The optimal artificial neural network obtained by the repeated structuring and learning procedure (RSL_ANN). It presents a three hidden layer architecture with 6 neurons in the first hidden layer, 6 neurons in the second hidden layer and 5 neurons in the third hidden layer.

**Figure 3:**
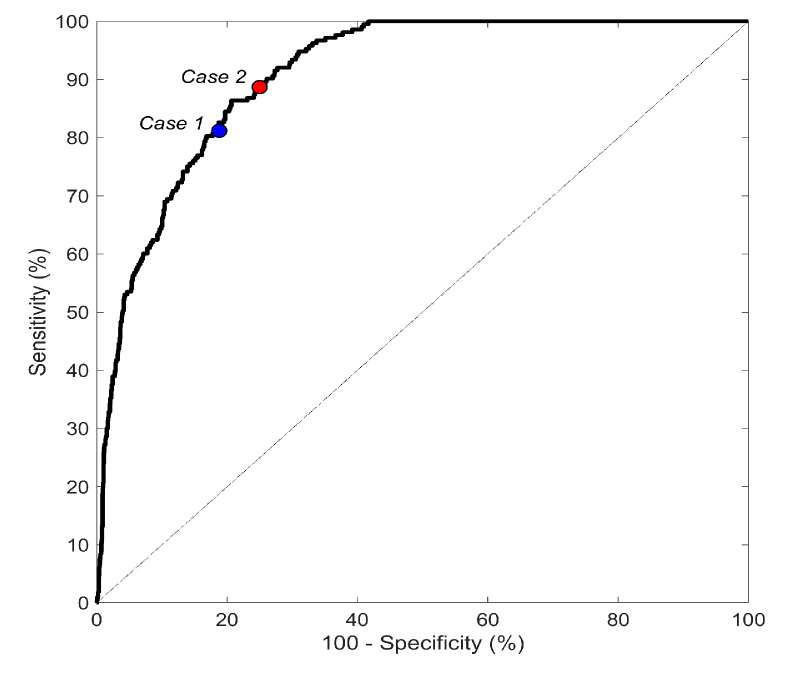
Receiving operating characteristic (ROC) for the testing dataset. The area under the curve (AUC) value is 90.8%. Operating points for Case 1 (blue dot), in which sensitivity (Se) and specificity (Sp) are both equal to 81.2%, and Case 2 (red dot), in which Sp is 75% and Se is 88.7%, are also reported.

The optimal RSL_ANN had a three hidden layer architecture with 6 neurons in the first hidden layer, 6 neurons in the second hidden layer and 5 neurons in the third hidden layer (Figure 2). The ROC curves for the testing dataset obtained with optimal RSL_ANN are depicted in Figure 3. The AUCs for the training, validation and testing datasets are 91.1% (CI: 89.1–93.0%), 90.2% (CI: 86.2–94.3%), and 90.8% (CI: 88.1–93.5%), respectively. Case 1 was characterized by values of Se and Sp both equal to 81.2% in the testing dataset. Eventually, Case 2 was characterized by a value of Sp equal to 75.0% and a value of Se equal to 88.7% in the testing dataset.

## 4. Discussion

This work proposes RSL_ANN as a supervised fully connected artificial neural network created using the repeated structuring and learning procedure [21] for reliable AF identification in ECGs acquired with the portable KARDIA device by AliveCor, as those used here and available in the Physionet “AF Classification from a Short Single Lead ECG Recording” database [19,22]. The repeated structuring and learning procedure has to be considered as a general method to construct ANNs and not in association with a specific clinical application. The used innovative repeated structuring and learning procedure [21] is indeed particularly suitable for applications of neural networks to relatively small databases (and not only to big data, as is usually done) since improving the loss function by iteratively alternating structuring and learning phases during the training (activation functions are standard).

RSL_ANN was fed with a set of 19 input features automatically extracted from ECGs (Table 1). By considering the three criteria for AF diagnosis, the features set includes standard morphological features of ECG waves (to reflect possible P-wave disappearance) as well as ECG features that typically characterize AF, that are F-waves features (to reflect possible F-waves appearance) and HRV features (to reflect possible HRV increment). Statistical analysis of feature distributions (Table 3) confirmed the known clinical observations that, in AF, the P wave disappears, F waves appear and HRV increases. P-wave disappearance and F-waves appearance are indicated by the finding that AP values are significantly higher in the non-AF class than the AF class. AP values in the AF class are not 0 (as one would expect in the absence of the P wave) because of representing F-waves amplitude and not the P -wave amplitude (see Section 2.2). F-waves appearance in AF is also indicated by the fact that all FWFR features were significantly higher in the AF class than in the non-AF class. Finally, the HRV increment in AF is indicated by the fact that all HRV features were significantly higher in the AF class than in the non-AF class. These findings, together with the observation that only two morphological and not AF-specific features out of 19 (both related to the QRS complex) were not significantly different in AF vs. non-AF classes (Table 3), confirm the reliability of the automatic feature extraction and the appropriateness of the feature selection.

RSL_ANN output is the ECG classification score, that is a value between 0 (indicating a subject not affected by AF) and 1 (indicating a subject affected by AF). No further stratification for cardiac rhythms other than AF was provided for the non-AF cases since optimal identification of a specific cardiac rhythm or pathology requires a specifically designed artificial neural network and proper selection of input ECG features (for example, in [21,32] optimal artificial neural networks for identification of heart failure in post-infarction patients and of ischemia in patients who underwent elective percutaneous coronary intervention are proposed, both obtained using the repeated structuring and learning procedure and a different set of 13 input ECG features).

As said, use of ECG features instead of raw data (as sometimes done when using long short-term memory, 1D convolutional neural network and others [11,15,16,33,34]) at the input of RSL_ANN implies adding an ECG processing step for feature extraction before classification; however, it also allows the construction of a faster and simpler artificial neural network, since based on a reduced number of hidden layers, through a smaller training dataset. In addition, since each feature, if well selected, reflects a specific physiologic phenomenon, classification logic of a network is physiologically more understandable than when it is based on raw data, and this is very much appreciated in context in which interpretability of the model is desirable.

RSL_ANN was constructed and tested on the “AF Classification from a Short Single Lead ECG Recording” database [19] by Physionet [22]; this database was selected for several reasons. First, it contains more than 8000 short single-lead ECG recordings and thus represents a suitable database for the design of a tool based on artificial neural networks. Additionally, these ECGs were acquired using the KARDIA [19], which is a portable device by AliveCor, in healthy subjects and patients showing several types of cardiac rhythm besides AF. These characteristics of the database allowed us to test the proposed algorithm in relation to the two main confounders in automatic AF identification, which are the level of noise affecting ECGs acquired using portable devices and the presence of arrhythmias other than AF.

Less than 3% of the ECGs included in the database could not be used in this study due to high levels of noise that jeopardized R-peak detection, and thus not for issues related to feature extraction or RSL_ANN construction. Nevertheless, all the observations that can be done on RSL_ANN ability to identify AF hold for ECGs affected by various levels of noise but in which the signal is dominant with respect to noise. Reliability of RSL_ANN in very noisy conditions remains to be demonstrated and requires availability of an R-peak detector able to perform correctly in such adverse conditions. Performance of RSL_ANN was very good and very similar in all datasets, with AUC over 90%. This result confirms the ability of RSL_ANN to correctly generalize the problem of AF identification. We made the choice to express RSL_ANN performance in terms of AUC in order to optimize Se and Sp (and thus working points in ROC and the threshold value of an output neuron) according to applications. When RSL_ANN is applied to a subject with no history of AF, errors in AF and non-AF classifications should be equally probable. Consequently, the threshold should be chosen to have equal values of Se and Sp. This case corresponds to Case 1, in which Se and Sp are 81.2% in the testing dataset. Instead, if RSL_ANN is applied to a subject with history of AF, AF occurrence is more likely and errors in AF identification should be minimized with respect to errors in non-AF identifications. Consequently, the threshold should be chosen to have the maximum Se obtainable by setting Sp at its minimum acceptable value. This case corresponds to Case 2 in which a Se of 88.7% is obtained by setting Sp at 75.0%.

Comparison of our RSL_ANN performance against that of other studies in the literature is not a straightforward task due to the many differences among them. Firstly, some studies used data acquired with traditional ECG recorders [11,12,13,14,15,16,18] and some others with portable devices [5,6,7,8,9,10,17]. Recorded signals included ECGs [11,12,13,14,15,16,17,18] but also photoplethysmograms [5,6,7,8,9,10]. Moreover, some studies performed intra-subject AF episodes identification within an ECG [11,15,18], others identified ECGs with at least one AF episode among patients [5,6,7,8,9,10,12,13,14,16,17]. Only a few studies took into account the main AF confounders, which are other abnormal rhythms [12,16,17,18] or noises [10,16]. All studies relied on their own selected features; most studies considered features related to HRV [5,6,7,8,9,10,13,14,18], few studies also included features related to ECG morphology [12,17], and no study but ours considered features related to Fwaves. Some studies directly considered ECG time-sequences (instead of features) as classifier input [11,15,16]. Different types of classifiers were proposed, among which the standard statistical comparison [5,6,7,8,9], the support vector machine [10,14,17], the convolutional neural network [11,15], the ANN [12,18], the XGBoost classifier [13], the modified Elman neural network [15] and the hierarchical extreme learning machine [16]. Finally, most studies reported only values of Se and Sp (higher than 90%) [5,6,7,9,10,11,12,13,14,15,16]; few of them also reported values of AUC [5,13]. Despite the several observed differences among studies, an attempted qualitative comparison is reported in Table 4.

Our RSL_ANN uses the highest number of data acquired by a portable device, considers all main confounders in AF identification and uses all AF diagnosis features. Some studies report values of Se (>90%) or AUC (>90%) higher than ours but involved discrimination of clinical ECGs (acquired with medical machines such as electrocardiograph or Holter ECG) [11,12,13,14,15,16,18] showing AF rhythm from clinical ECGs showing normal sinus rhythm only [5,6,7,8,9,10,11,13,14,15]. These working conditions are much easier than those considered in this study, in which ECGs were acquired by a portable device and discrimination of AF rhythms is not only from normal sinus rhythm but also from other arrhythmias. One work [17] created a classifier able to detect AF using the same database of our paper and obtained values of Se and Sp equal to 77.5% and 97.9%, respectively; thus, differently from us, it made the choice to optimize Sp over Se. In any event, we believe that the ROC curve should always be provided since the choice of a threshold is for the specialists in medical decision making. Finally, some proceedings from Computing in Cardiology 2017 used the same database as training dataset, but then validated their methods in another dataset, which however is not open-source available. Considering this discrepancy, a comparison between these studies and our work would be biased.

Eventually, reliable identification of AF in ECG acquired by portable or wearable devices is important for large scale preventive screening among the entire worldwide population [35]. In this context, our RSL_ANN represents a reliable software application to be associated to one of them to contrast the socio-economic repercussions related to AF due to its usual late diagnosis. Future studies are needed to definitely validate the use of the RSL_ANN for large scale AF screening.

## 5. Conclusions

Our proposed supervised fully connected artificial neural network created using the repeated structuring and learning procedure was able to reliably identify atrial fibrillation from the data acquired with the portable KARDIA device by AliveCor available in the Physionet “AF Classification from a Short Single Lead ECG Recording” database. Thus, our proposed artificial neural network represents a promising tool for a reliable identification of atrial fibrillation from ECGs acquired by portable devices, even when affected by other abnormal rhythms and corrupted by noise.

## Figures and Tables

**Table 1:** Summary of the 19 ECG features (11 on morphology, 4 on F waves and 4 on heart-rate variability) characterizing each ECG recording.

Feature Type	Feature Name	Feature Unit	Feature Description
Morphology	P_p_R_p_	ms	time interval between P_P_ and R_P_
	P_p_QRS_off_	ms	time interval between P_P_ and QRS_off_
	QRS_on_QRS_off_	ms	time interval between QRS_on_ and QRS_off_
	QRS_on_T_off_	ms	time interval between QRS_on_ and T_off_
	QRS_off_T_off_	ms	time interval between QRS_off_ and T_off_
	AP	µV	amplitude of the MECGB at P_P_
	AQRS_on_	µV	amplitude of the MECGB at QRS_on_
	AQRS	µV	max-min of MECGB amplitude between QRS_on_ and QRS_off_
	AQRS_off_	µV	amplitude of the MECGB at QRS_off_
	AT	µV	amplitude of the MECGB at T_P_
	AQRS/AP	dimensionless	ratio between AQRS and AP
Fwaves	FWFR_FFT_	%	Fast Fourier Transform spectral ratio
	FWFR_WLC_	%	Welch’s method spectral ratio
	FWFR_YWK_	%	Yule-Walker’s method spectral ratio
	FWFR_THM_	%	Thomson’s method spectral ratio
Heart-rate variability	MRR	ms	mean RR interval
	SDRR	ms	RR-interval standard deviation
	RMSRR	ms	Root mean square of RR interval
	PRR50	%	% of RR > previous RR of more than 50 ms

**Table 2:** Data division into training dataset, validation dataset and testing dataset.

	ALL	TRAINING DATASET	VALIDATION DATASET	TESTING DATASET
**AF**	707	395	99	213
**Non-AF**	7321	4098	1026	2197
**TOTAL**	8028	4493	1125	2410

**Table 3:** Feature distributions of both AF and non-AF of all data, training, validation and testing datasets.

		ALL DATA		TRAINING		VALIDATION		TESTING	
				DATASET		DATASET		DATASET	
		AF	Non-AF	AF	Non-AF	AF	Non-AF	AF	Non-AF
**Morphological Features**	**P_p_R_p_**	207 *	150	203 *	150	197 *	150	220 *	150
	**(ms)**	[161;243]	[130;183]	[157;240]	[130;180]	[153;237]	[130;187]	[183;247]	[130;183]
	**P_p_QRS_off_**	257 *	200	250 *	200	250 *	200	267 *	200
	**(ms)**	[210;287]	[177;233]	[203;287]	[177;233]	[203;286]	[177;240]	[227;293]	[179;233]
	**QRS_on_QRS_off_**	103	103	100	103	103	103	103	103
	**(ms)**	[93;113]	[93;113]	[93;113]	[93;113]	[90;113]	[93;113]	[93;113]	[93;113]
	**QRS_on_T_off_**	333 *	386	330 *	387	337 *	383	333 *	383
	**(ms)**	[261;387]	[320;427]	[260;383]	[323;427]	[276;399]	[313;423]	[259;407]	[317;427]
	**QRS_off_T_off_**	230 *	283	223 *	287	240 *	280	230 *	283
	**(ms)**	[157;283]	[217;320]	[150;277]	[220;320]	[178;290]	[213;320]	[153;301]	[213;320]
	**AP**	12 *	52	13 *	52	12 *	49	−10 *	55
	**(µV)**	[−25;34]	[−34;82]	[−25;37]	[−37;82]	[−26;38]	[−36;80]	[−24;26]	[−27;83]
	**AQRS_on_**	0 *	−5	0 *	−5	0 *	−4	1 *	−4
	**(µV)**	[−7;7]	[−17;4]	[−8;6]	[−18;4]	[−5;7]	[−16;4]	[−5;9]	[−17;4]
	**AQRS**	852 *	895	852	894	873	873	836 *	905
	**(µV)**	[637;1075]	[651;1158]	[664;1075]	[646;1533]	[615;1092]	[636;1140]	[631;1062]	[670;1175]
	**AQRS_off_**	−27	−24	−29 *	−22	−16	−22	−28	−28
	**(µV)**	[−73;9]	[−64;13]	[−75;8]	[−63;13]	[−55;16]	[−62;15]	[−76;9]	[−67;11]
	**AT**	185 *	246	180 *	248	195 *	236	188 *	247
	**(µV)**	[109;259]	[165;332]	[109;253]	[167;334]	[127;253]	[156;319]	[105;269]	[167;336]
	**AQRS/AP**	−3 *	9	−1 *	9	−3 *	9	−7 *	9
	**(dimension-less)**	[−24;20]	[−1;13]	[−24;19]	[−2;13]	[−26;15]	[−1;13]	[−23;23]	[−1;14]
**F-Waves Features**	**FWFR_FFT_**	24 *	14	23 *	14	25 *	14	23 *	15
	**(%)**	[16;31]	[9;21]	[16;30]	[9;21]	[16;31]	[9;21]	[16;31]	[10;21]
	**FWFR_WLC_**	25 *	14	25 *	14	25 *	14	24 *	15
	**(%)**	[17;32]	[9;21]	[17;32]	[9;21]	[16;32]	[10;22]	[17;32]	[10;21]
	**FWFR_YWK_**	35 *	23	35 *	23	37 *	22	34 *	23
	**(%)**	[25;45]	[17;31]	[26;45]	[17;31]	[25;44]	[16;31]	[24;43]	[17;31]
	**FWFR_THM_**	24 *	14	24 *	14	25 *	14	23 *	14
	**(%)**	[16;31]	[9;21]	[16;32]	[9;21]	[16;31]	[9;21]	[16;31]	[10;21]
**HRV Features**	**MRR**	712 *	864	692 *	862	717 *	869	755 *	863
	**(ms)**	[580;860]	[758;976]	[565;835]	[760;979]	[577;878]	[751;980]	[616;902]	[758;970]
	**SDRR**	157 *	57	155 *	58	157 *	58	163 *	54
	**(ms)**	[104;224]	[24;134]	[101;208]	[25;136]	[101;227]	[24;133]	[112;242]	[22;129]
	**RMSRR**	218 *	57	215 *	59	223 *	56	223 *	52
	**(ms)**	[144;309]	[19;172]	[138;299]	[20;174]	[142;319]	[19;170]	[159;320]	[18;167]
	**PRR50**	92 *	67	93 *	67	93 *	67	93 *	67
	**(%)**	[90;94]	[0;83]	[90;94]	[0;83]	[90;94]	[0;83]	[89;94]	[0;80]

* *p*-value < 0.05 when comparing corresponding feature in AF vs. non-AF classes, within a dataset.

**Table 4:** Comparison between our work and the literature.

Reference	Data acquisition	Confounders	Input	Classifier	AUC	Se	Sp
[5]	Portable devices (iPhone 4S); 120 PPGs	Not considered	HRV features	Statistical comparison	93.1	95.0	95.0
[6]	Portable devices; 242 PPGs	Not considered	HRV features	Statistical comparison	Not reported	98.0	88.0
[7]	Portable devices (iPhone); 97 PPGs	Not considered	HRV features	Statistical comparison	Not reported	93.1	90.1
[8]	Portable devices (iPhone); 88 PPGs	Not considered	HRV features	Statistical comparison	Not reported	66.6	78.9
[9]	Portable devices (iPhone 4S); 25 PPGs	Not considered	HRV features	Statistical comparison	Not reported	97.6	99.6
[10]	Portable devices (Sony Xperia); 16 PPGs	Noise	HRV features	SVM	Not reported	93.8	100
[11]	Holter ECG recorders; 139 ECGs	Not considered	ECG time sequence	CNN	Not reported	99.2	98.7
[12]	ECG recorders; 2363 ECGs	Other abnormal rhythms	Morphological and HRV features	ANN	Not reported	89.9	92.8
[13]	Holter ECG recorders; 1656 ECGs	Not considered	HRV features	XGB	98.9	98.4	99.5
[14]	Atrial ECG recorder; 113 ECGs	Not considered	HRV features	SVM	Not reported	99.9	96.6
[15]	Holter ECG recorders; 23 ECGs	Not considered	ECG time sequence	CNN + MENN	Not reported	97.9	97.1
[16]	ECG recorders; 47 ECGs	Other abnormal rhythms	ECG time sequence	HELM	Not reported	98.77	100
[17]	Portable Devices (KARDIA by AliveCor); 8244 ECGs	Other abnormal rhythms and noise	Morphological and HRV features	SVM	Not reported	77.5	97.9
[18]	ECG recorders; 12 ECGs	Other abnormal rhythms	HRV features	ANN	Not reported	84.9	75.4
This work	Portable Devices (KARDIA by AliveCor); 8244 ECGs	Other abnormal rhythms and noise	Morphological, F-waves and HRV features	ANN	90.8	Case1: 81.2 Case2: 88.7	Case1: 81.2 Case2: 75.0

ANN: artificial neural network; AUC: area under the curve; CNN: convolutional neural network; ECG: electrocardiogram; HELM: hierarchical extreme learning machine; HRV: heart-rate variability; MENN: modified Elman neural network; PPG: photoplethysmogram; Se: sensitivity; Sp: specificity; SVM: support vector machine; XGB: XGBoost classifier.
